# Supplementary material for: Transcriptomic and Metatranscriptomic Analyses Provide New Insights into the Response of the Pea Aphid Acyrthosiphon pisum (Hemiptera: Aphididae) to Acetamiprid
Source: Insects. 2024 Apr 15;15(4):274. doi: 10.3390/insects15040274 (PMC11050337; doi:10.3390/insects15040274)
Supplement: Supplementary file 1 [file insects-15-00274-s001.zip › insects-2947461-supplementary.pdf]

Supplementary Table S1 Summary of transcriptomic sequencing data

Sample	Total Raw Reads (M)	Total Clean Reads (M)	Total Clean Bases (Gb)	Clean Reads Q20 (%)	Clean Reads Q30 (%)	Clean Reads Ratio (%)
RS_1	45.57	42.73	6.41	97.67	93.71	93.76
RS_2	45.57	43.33	6.5	97.34	92.81	95.07
RS_3	45.57	43.34	6.5	97.28	92.72	95.09
SS_1	45.57	42.47	6.37	97.05	92.39	93.19
SS_2	45.57	42.16	6.32	97.09	92.53	92.5
SS_3	47.33	42.62	6.39	97.02	92.35	90.06

Supplementary Table S2 Summary of metatranscriptomic sequencing data

Sample	Raw reads	Clean reads	Clean bases	Q20 (%)	Q30 (%)	GC (%)
RS_1	240762172	240762172	24055600772	100	100	53.29
RS_2	240883554	240883554	24058671924	100	100	52.86
RS_3	240268794	240268794	24006344364	100	100	52.53
SS_1	240545312	240545312	24029416710	100	100	51.56
SS_2	240341504	240341504	24003051416	100	100	52.15
SS_3	240284986	240284986	24011438320	100	100	52.81
